# Anti-Nogo-A Immunotherapy Does Not Alter Hippocampal Neurogenesis after Stroke in Adult Rats

**DOI:** 10.3389/fnins.2016.00467

**Published:** 2016-10-18

**Authors:** Daniel J. Shepherd, Shih-Yen Tsai, Timothy E. O'Brien, Robert G. Farrer, Gwendolyn L. Kartje

**Affiliations:** ^1^Neuroscience Institute, Loyola University Chicago Health Sciences DivisionMaywood, IL, USA; ^2^Research Service, Edward Hines Jr. VA HospitalHines, IL, USA; ^3^Department of Mathematics and Statistics, Loyola University ChicagoChicago, IL, USA; ^4^Department of Molecular Pharmacology and Therapeutics, Loyola University Chicago Health Sciences DivisionMaywood, IL, USA

**Keywords:** stroke, neurogenesis, Nogo-A, immunotherapy, myelin-associated inhibitor

## Abstract

Ischemic stroke is a leading cause of adult disability, including cognitive impairment. Our laboratory has previously shown that treatment with function-blocking antibodies against the neurite growth inhibitory protein Nogo-A promotes functional recovery after stroke in adult and aged rats, including enhancing spatial memory performance, for which the hippocampus is critically important. Since spatial memory has been linked to hippocampal neurogenesis, we investigated whether anti-Nogo-A treatment increases hippocampal neurogenesis after stroke. Adult rats were subject to permanent middle cerebral artery occlusion followed 1 week later by 2 weeks of antibody treatment. Cellular proliferation in the dentate gyrus was quantified at the end of treatment, and the number of newborn neurons was determined at 8 weeks post-stroke. Treatment with both anti-Nogo-A and control antibodies stimulated the accumulation of new microglia/macrophages in the dentate granule cell layer, but neither treatment increased cellular proliferation or the number of newborn neurons above stroke-only levels. These results suggest that anti-Nogo-A immunotherapy does not increase post-stroke hippocampal neurogenesis.

## Introduction

Cognitive impairment is a recognized sequela of ischemic stroke (Gottesman and Hillis, 2010). Our laboratory has previously shown that treatment with function-blocking antibodies against the neurite growth-inhibitory protein Nogo-A (anti-Nogo-A immunotherapy) improves spatial memory performance after stroke in aged rats (Gillani et al., 2010), but a cellular mechanism of efficacy has not yet been identified. We and others have previously demonstrated that anti-Nogo-A immunotherapy stimulates dendritic and axonal remodeling and increases dendritic spine density in the contralesional sensorimotor cortex after stroke (Papadopoulos et al., 2002, 2006; Wiessner et al., 2003; Seymour et al., 2005; Tsai et al., 2007, 2011; Lindau et al., 2014). These neuroplastic changes may underlie the sensorimotor recovery seen in anti-Nogo-A treated animals (Papadopoulos et al., 2002, 2006; Wiessner et al., 2003; Seymour et al., 2005; Tsai et al., 2007, 2011; Lindau et al., 2014; reviewed by Kumar and Moon, 2013), as silencing of newly sprouted axonal connections ablates the sensorimotor recovery promoted by anti-Nogo-A treatment (Wahl et al., 2014). However, no changes in dendritic complexity or spine density were found in anti-Nogo-A-treated animals in pyramidal neurons of CA1 or CA3 or in dentate granule cells, despite spatial memory improvement, suggesting an alternate mechanism of efficacy (Gillani et al., 2010). We and other groups have likewise reported that anti-Nogo-A treatment enhances recovery from hemispatial neglect after aspiration lesion of the medial agranular cortex (Brenneman et al., 2008) and recovery of cognitive function after traumatic brain injury (Lenzlinger et al., 2005; Marklund et al., 2007), positioning Nogo-A as a promising therapeutic target for improving cognition after brain injury.

Nogo-A is a transmembrane protein with two main inhibitory domains (Nogo-66 and Nogo-A-Δ20), and acts primarily by activating two different cell surface receptors. Nogo-66 binds to the Nogo receptor NgR1, leading to activation of the small GTPase RhoA and subsequent activation of Rho-associated protein kinase (ROCK). Nogo-A-Δ20 activates the previously characterized sphingosine-1-phosphate receptor S1PR2, which also activates RhoA/ROCK and may also influence gene expression. Both receptors have been found to play a role in mediating structural and synaptic plasticity (Schwab and Strittmatter, 2014).

Several studies have linked hippocampal neurogenesis and spatial memory performance on the Morris water maze (reviewed by Garthe and Kempermann, 2013), and interventions that increase neurogenesis have also been shown to improve Morris water maze performance after brain injury, including stroke (Wurm et al., 2007; Meng et al., 2014). Whether Nogo-A plays a direct role in adult hippocampal neurogenesis is unknown. However, a previous study reported that mice deficient for the Nogo receptor NgR1 exhibit increased hippocampal neurogenesis and reduced cognitive impairment after traumatic brain injury (Tong et al., 2013). Furthermore, at the molecular level, the key Nogo-A signaling mediators RhoA and ROCK play a suppressive role in hippocampal neurogenesis (Keung et al., 2011; Christie et al., 2013; reviewed by Vadodaria and Jessberger, 2013). Nogo-A signaling has also been shown to inhibit nerve growth factor-mediated CREB phosphorylation *in vitro* (Joset et al., 2010), whereas CREB phosphorylation is important for the maturation and survival of newborn dentate granule cells, including after stroke (Zhu et al., 2004; Jagasia et al., 2009). These studies raise the question of whether antibody-mediated Nogo-A neutralization could lead to alterations in neurogenesis, which may in turn contribute to cognitive recovery after stroke.

The goal of this study was to determine whether Nogo-A neutralization enhanced post-stroke hippocampal neurogenesis. Our results showed that while infusion of both anti-Nogo-A and control antibodies led to the accumulation of new microglia/macrophages in the hippocampus, Nogo-A neutralization did not affect the number of newborn neurons in the dentate gyrus after stroke. Therefore, enhanced neurogenesis is unlikely to contribute to the improvement in spatial memory that we previously reported after stroke and anti-Nogo-A immunotherapy.

## Materials and methods

### Animal subjects

All animal experiments were approved by the Institutional Animal Care and Use Committee of the Hines Veterans Affairs Hospital. A total of 42 adult male Long-Evans black hooded rats (Harlan, Indianapolis, IN), 12 weeks of age at study initiation, were used. See Table 1 for an overview of experimental design. Animals were housed in pairs on a 12 h light-dark cycle with *ad lib* food and water.

**Table 1:** **Overview of experimental groups**.

	***N***	**Treatment duration**	**BrdU**	**Sacrifice**
**PROLIFERATION**				
Stroke only	6	None	200 mg/kg i.p. on day 21 post-stroke	2 h after BrdU injection
Stroke/Control Ab	6	14 days		
Stroke/Anti-Nogo-A Ab	6	14 days		
**DIFFERENTIATION/SURVIVAL**				
Stroke only	8	None	50 mg/kg twice/day for 5 days beginning day 7 post-stroke	8 weeks post-stroke
Stroke/Control Ab	5	14 days		
Stroke/Anti-Nogo-A Ab	8	14 days		

### Middle cerebral artery occlusion

Rats were anesthetized with 2% isoflurane in oxygen. Distal middle cerebral artery occlusion was performed as described previously (Chen et al., 1986; Papadopoulos et al., 2002). An incision through the scalp and temporalis muscle was made, followed by a craniotomy to expose the middle cerebral artery (MCA). The left MCA was then ligated with 10-0 suture and bisected. After making a midline ventral neck incision, the left common carotid artery (CCA) was permanently ligated with 4-0 suture, and the right CCA was occluded for 1 h using an aneurysm clip. Body temperature was maintained at 37°C throughout the procedure by a thermoregulator and heating pad. After incisions were closed, animals were allowed to recover in their home cage. Sham surgery animals were anesthetized for an equivalent duration and given neck and scalp incisions.

### Anti-Nogo-A treatment antibody production and purification

The hybridoma cell line for the mouse monoclonal anti-Nogo-A antibody 11C7 was provided by Prof. Martin Schwab (Brain Research Institute, University of Zurich). The cells were grown in Hybridoma-SFM (Gibco, Waltham, MA) using the CELLLine multi-chamber cell cultivation system (BD Biosciences, San Jose, CA) according to manufacturer's protocol. The 11C7 antibody was purified from antibody-containing medium by Protein-G column chromatography (Pierce, Waltham, MA). Coomassie blue staining of purified antibody separated on denaturing polyacrylamide gels routinely showed only two bands corresponding to heavy and light chains. For infusion, purified 11C7 was diluted to 2.5 mg/mL in sterile phosphate-buffered saline.

### Intracerebroventricular antibody treatment

One week following stroke (a delay in treatment that still improves functional recovery, Seymour et al., 2005; Gillani et al., 2010), rats were anesthetized with isoflurane and implanted with a subcutaneous osmotic minipump (Alzet model 2ML2; Durect Corporation, Cupertino, CA) connected to a cannula leading to the ipsilesional lateral cerebral ventricle, as previously done. Either anti-Nogo-A mouse IgG1 (antibody 11C7) or a control antibody raised against a non-mammalian peptide (anti-cyclosporine A; mouse IgG1, a generous gift from Novartis International AG; Craveiro et al., 2013), both 2.5 μg/μL, were infused at a rate of 5 μL/h (as previously done, Markus et al., 2005; Gillani et al., 2010) for 14 days. At the end of the treatment period, pumps were removed under isoflurane anesthesia.

### 5-bromo-2′-deoxyuridine (BrdU) injections

BrdU (Sigma-Aldrich Co., St. Louis, MO) was dissolved at 20 mg/mL in sterile saline plus 0.007 N NaOH and sterilized by passing through a 0.22 μm syringe filter. Rats were injected intraperitoneally according to one of two injection schedules. To measure cellular proliferation, rats were injected with a single dose of 200 mg/kg body weight BrdU (a saturating dose, even in animals with increased hippocampal neurogenesis; Eadie et al., 2005) and killed 2 h after injection. For long-term phenotype analysis of proliferating cells, rats were injected with 50 mg/kg BrdU twice per day for 5 consecutive days, beginning 7 days after stroke.

### Tissue collection and preparation

Animals were euthanized by overdose with Euthasol (390 mg/kg i.p., Virbac, Fort Worth, TX) and transcardially perfused with cold heparinized saline followed by 4% paraformaldehyde (PFA). Brains were extracted and post-fixed overnight in 4% PFA, cryoprotected in 30% sucrose until sinking, and embedded in OCT on dry ice. 40 μm sections were cut using a Leica CM1850 cryostat and stored in cryoprotectant solution at −20°C until use.

### Histology

For BrdU immunostaining, tissue sections were mounted on plus-charged slides, dried at room temperature overnight, and then immersed in 99–100°C 10 mM sodium citrate pH 6 for 15 min (Tang et al., 2007). Slides were then placed in sodium phosphate buffer (PB) and sections carefully removed from the slides using a razor blade, allowing subsequent staining steps to be performed free-floating. Sections were then incubated in primary antibodies diluted in PB pH 7.4 plus 0.2% Tween 20 overnight at 4°C with gentle agitation. After extensive washing in PB/0.2% Tween-20, tissue was incubated in secondary antibody (conjugated to either biotin or fluorophores) diluted in PB/0.2% Tween-20 for 2 h at room temperature (see Table 2 for a list of antibodies and dilutions used in this study). S1PR2 immunostaining was detected by avidin-biotin peroxidase complex (VectaStain Elite ABC kit; Vector Laboratories, Burlingame, CA) followed by AlexaFluor 568 tyramide signal amplification (Thermo Fisher T20949) per manufacturer's instructions. For fluorescence microscopy, nuclei were counterstained with DAPI. For chromogenic detection, sections incubated in biotinylated secondary antibody were then incubated in avidin-biotin complex (Vector Laboratories) for 1 h and reacted in nickel-enhanced 3,3′diaminobenzidine (DAB, Sigma-Aldrich Co.). Fluorescent immunostained tissue was mounted on gelatin-subbed slides and coverslipped with Fluoromount G mounting media (Southern Biotech, Birmingham, AL). DAB tissue was mounted on gelatin-subbed slides, dehydrated in graded ethanols, cleared in xylene, and coverslipped with Permount (Fisher Scientific, Waltham, MA).

**Table 2:** **Antibodies used for immunofluorescence and immunohistochemistry**.

**Antibody**	**Source**	**Dilution**
**PRIMARY ANTIBODIES**		
Mouse IgG2a anti-BrdU	Pierce MA3-071 [RRID: AB_10986341]	1:1000–5000
Rabbit anti-doublecortin (DCX)	Cell Signaling 4604S [RRID: AB_10693771]	1:500
Goat anti-doublecortin (DCX)	Santa Cruz SC-8066 [RRID: AB_2088494]	1:500
Rabbit anti-GFAP	Dako Z0334 [RRID: AB_10013382]	1:1000
Mouse IgG1 anti-GFAP	Chemicon MAB360 [AB-11212597]	1:1000
Rabbit anti-Iba1	Wako 019-19741 [RRID: AB_839503]	1:5000
Rat anti-myelin basic protein (MBP)	Abcam Ab7349 [RRID: AB_305869]	1:100
Mouse IgG1 anti-NeuN	Chemicon MAB377 [RRID: AB_2298772]	1:1000
Rabbit anti-NeuN	Millipore ABN78 [RRID: AB_11211087]	1:1000
Rabbit anti-NgR1	Alomone Labs ANT-008 [RRID: AB_2040180]	1:250
Mouse IgG1 anti-Nogo-A	mAb 11C7 produced from hybridoma cell line	0.25 μg/mL
Human anti-S1PR2	AbD Serotec custom antibody AbD14533.1	1:50
Rabbit anti-Sox2	Abcam Ab97959 [RRID: AB_10013822]	1:1000
**SECONDARY ANTIBODIES**		
Goat anti-mouse (AlexaFluor 488)	ThermoFisher A11001 [RRID: AB_10566289]	1:1000
Goat anti-mouse IgG2a (AlexaFluor 488)	ThermoFisher A21131 [RRID: AB_141618]	1:1000
Goat anti-mouse IgG2a (biotinylated)	Jackson Immunoresearch 115-065-206 [RRID: AB_2338572]	1:1000
Donkey anti-mouse (rat serum protein adsorbed; biotinylated)	Jackson Immunoresearch 715-065-151 [RRID: AB_2340785]	1:1000
Goat anti-mouse IgG1 (AlexaFluor 568)	ThermoFisher A21124 [RRID: AB_141611]	1:1000
Donkey anti-mouse (rat serum protein adsorbed; DyLight 488)	Jackson Immunoresearch 715-486-151 [RRID: AB_2572300]	1:200
Goat anti-rabbit (AlexaFluor 568)	ThermoFisher A11036 [RRID: AB_143011]	1:1000
Goat anti-rabbit (AlexaFluor 647)	ThermoFisher A21244 [RRID: AB_142672]	1:1000
Donkey anti-goat (AlexaFluor 488)	ThermoFisher A11055 [RRID: AB_2534102]	1:1000
Goat anti-human (biotinylated)	BioRad STAR126B [RRID: AB_961503]	1:500
Donkey anti-rat (AlexaFluor 594)	ThermoFisher A21209 [RRID: AB_10562899]	1:500

### Quantification

All quantification was performed by an investigator blind to experimental group.

#### Cellular proliferation

Six 40 μm sections per subject (*n* = 6 per group) encompassing the dorsal DG (every 12th section beginning at the rostral appearance of the dentate granule cell layer, between −2 and −4.8 mm with respect to bregma; Paxinos and Watson, 1998) were immunostained for BrdU and examined using bright-field microscopy on a Leica DM4000B microscope with a 40x/0.75 NA objective. Exhaustive cell counts were performed by manually counting all BrdU+ nuclei in the subgranular zone (SGZ) and basal layers of the granule cell layer (GCL) (within approximately 3 nuclei from the interface between the dentate granule cell layer and polymorphic layer) of the dorsal hippocampus bilaterally. Cell counts were multiplied by 12 to estimate the total number of proliferating cells.

#### BrdU+ cell counts at 8 weeks post-stroke

Six 40 μm sections (every 12th section beginning at the rostral appearance of the dentate granule cell layer; stroke/control antibody: *n* = 5; stroke only and stroke/anti-Nogo-A antibody: *n* = 8) were stained for BrdU, lightly counterstained with toluidine blue to identify the GCL, mounted, and coverslipped. BrdU+ nuclei within the dorsal DG GCL of each section were exhaustively counted using a 40x/0.75 NA objective and multiplied by 12 to estimate the total number of BrdU+ cells. Counts were then normalized to GCL volume using Cavalieri's principle (see below).

#### Measurement of GCL volume

The toluidine blue-stained tissue sections used for measuring total BrdU+ cells at 8 weeks post-stroke (6 sections total per subject) were imaged using MBF StereoInvestigator software. The Cavalieri Estimator probe was applied to measure GCL area and estimate the total volume of the GCL within the dorsal DG encompassed by the six sections.

#### Quantification of newborn cell phenotypes

A total of three 40 μm sections per subject (every 24th section beginning at the rostral appearance of the GCL [bregma −2 mm] and proceeding caudally) were stained for BrdU plus NeuN, Iba1, or Sox2, counterstained with DAPI, and examined on a Leica SPE confocal microscope using a 63x/1.3 NA oil immersion objective.

Due to the dense cellularity of the GCL and poor penetration of the NeuN antibody that confounded co-expression analysis in the middle of the tissue section, analysis of BrdU/NeuN co-labeling was restricted to near the outer surfaces of the tissue where NeuN expression was unambiguous. Approximately 50 cells per dentate gyrus per side were examined in each subject. Workflow was as follows: BrdU-positive cells were identified by first scanning the tissue with the appropriate excitation laser until positive nuclei within the GCL were identified. Then a single optical section was acquired with 1 Airy unit pinhole size, and channels merged to identify (1) total BrdU+ cells, and (2) the number of BrdU+ cells that were positive for either NeuN, Iba1, or Sox2. When co-labeling was not clear from a single optical section, z-stacks were acquired to disambiguate the labels.

Estimates of the total numbers of new neurons were calculated by multiplying the total number of BrdU+ cells by the proportion of BrdU+ cells expressing each marker.

#### Treatment antibody distribution and fluorescence intensity

Infused treatment antibody was detected using either a chromogen (DAB) or a fluorescent secondary antibody. For chomogenic detection, sections were incubated in a biotinylated anti-mouse IgG secondary antibody (rat serum protein adsorbed) overnight at 4°C (1:1000 in sodium phosphate buffer plus 0.3% Triton X100), followed by incubation in avidin-biotin peroxidase complex (Vector) and reaction in nickel-enhanced DAB. For visualization of the reaction product, the staining intensities of scanned tissue sections were then remapped in ImageJ (Schindelin et al., 2012) using the “Fire” look up table.

For fluorescence intensity analysis, three tissue sections through the dorsal DG (a 1 in 24 series) from an untreated, 7-day treated (3 subjects each from control antibody and anti-Nogo-A groups) and 8 weeks post-stroke (3 subjects each from control antibody and anti-Nogo-A groups) were washed in sodium phosphate buffer (PB) and incubated in DyLight-488-conjugated donkey anti-mouse (rat serum protein adsorbed) secondary antibody (Jackson Immunoresearch, West Grove, PA; 1:200 in PB/0.3% Triton X100) for 90 min at room temperature. Sections were washed in PB and then mounted on gelatin-subbed slides and coverslipped in Fluoromount G. Z stacks through the entire thickness of each tissue section were acquired using a 10x objective on a Leica SPE confocal microscope at equivalent parts of the DG in each tissue section. All image acquisition settings were kept constant. Image stacks were imported into ImageJ and compressed to maximum intensity Z projections. The mean gray value of the tissue was then measured in each section using ImageJ and averaged to yield a single intensity value for each hippocampus per subject. The tissue from the untreated (stroke-only) subject was used to determine background fluorescence, which is a combination of tissue autofluorescence and any potential non-specific binding of the fluorescent anti-mouse secondary antibody.

#### Lesion analysis

For each subject, a 1 in 24 tissue section series throughout each brain (excluding olfactory bulbs and cerebellum) was mounted on gelatin-subbed slides and stained with toluidine blue. Slides were then scanned at high resolution using a flatbed scanner and imported into Adobe Photoshop CS3, where the number of pixels in the intact and lesioned hemispheres was measured. To compute a lesion size as a percentage of the intact hemisphere, the total number of pixels in the lesioned hemisphere was subtracted from the total number of pixels in the intact hemisphere, and divided by the total intact hemisphere pixel number. This method therefore calculates the size of the missing, lesioned tissue.

### Statistics

Statistical analysis was performed using Minitab 17 and SAS 9.4 software. When appropriate, data were analyzed using one-way ANOVA, equal-variance *t*-tests or paired *t*-tests; when assumptions were not supported, unequal-variance ANOVA or *t*-tests or non-parametric tests (Wilcoxon rank-sum or Kruskal-Wallis) were used. Details regarding significance testing for each experiment can be found in Supplementary Table 1.

## Results

### Nogo-A is expressed by immature neurons in the normal adult dentate gyrus (DG)

**Figure 1:**
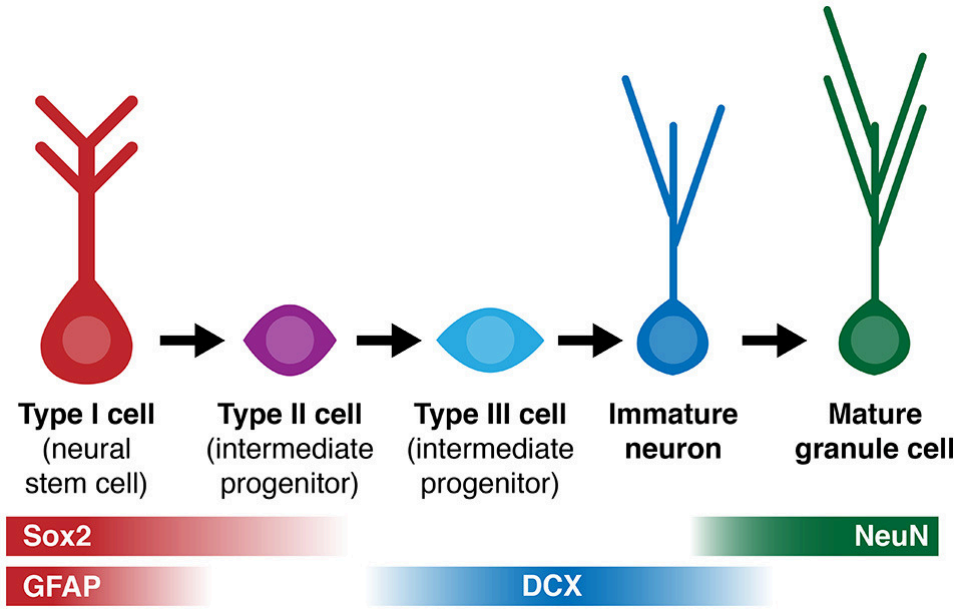
**Simplified diagram of cell lineage progression and stage-specific expression of markers (Sox2, GFAP, DCX, NeuN) referenced in this study**.

**Figure 2:**
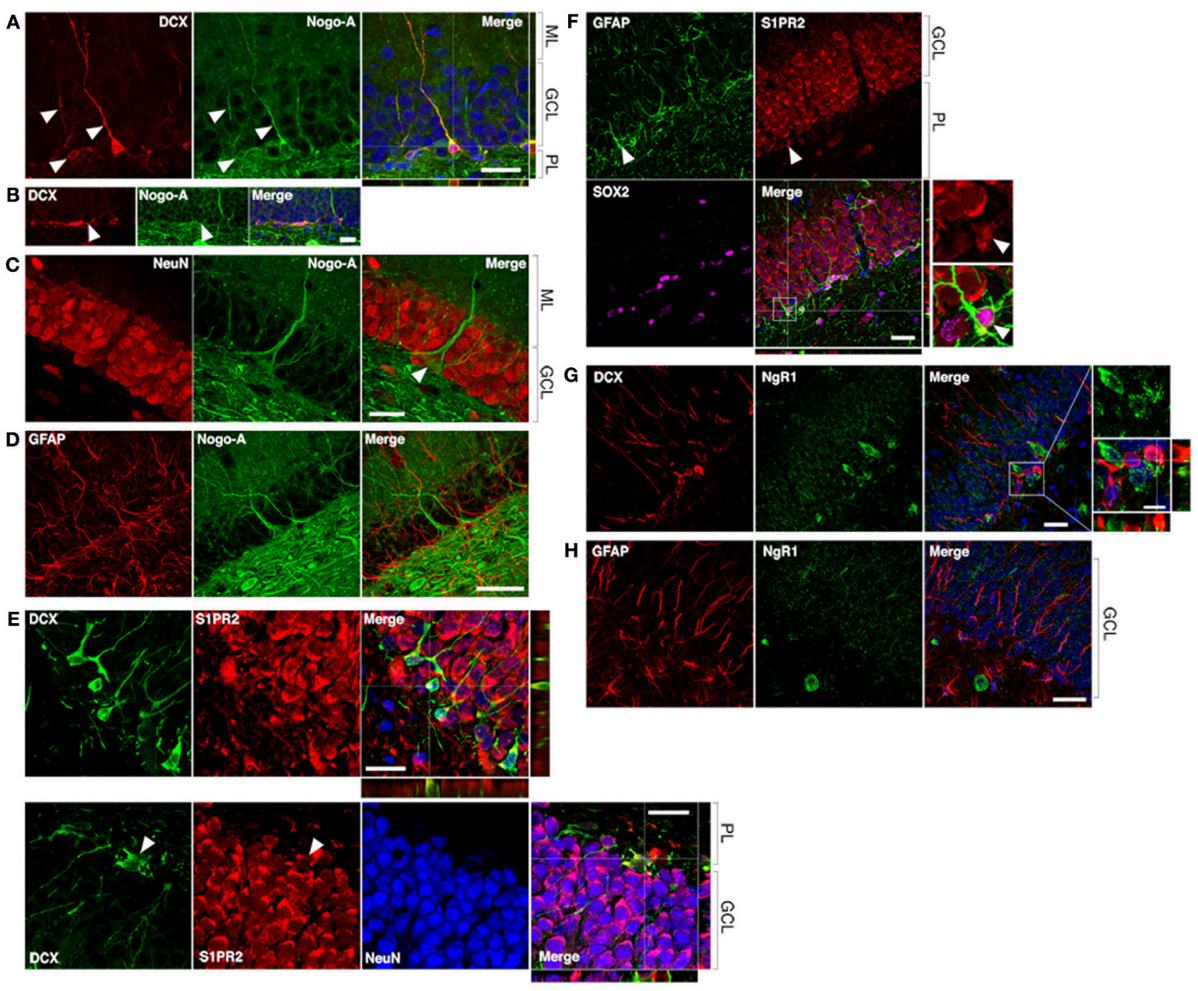
**Nogo-A is expressed by immature neurons in the adult dentate gyrus. (A)** Nogo-A is expressed in the processes and somata of immature neurons (arrowheads), which are also positive for doublecortin (DCX). Scale bar: 25 μm. **(B)** Horizontally-oriented DCX+/Nogo-A+ neuroblast. Scale bar: 25 μm. **(C)** Nogo-A expression is not appreciable in NeuN+ mature granule cells, the majority of NeuN+ cells in the GCL. However, putative basket cells (arrow) label strongly for Nogo-A. Scale bar: 25 μm. **(D)** Nogo-A immunoreactivity is not detectable in GFAP+ stem cells or astrocytes. Scale bar: 50 μm. **(E)** S1PR2 is broadly expressed in the GCL, including DCX+ immature neurons and NeuN+ mature neurons. Scale bar: 20 μm. **(F)** S1PR2 expression by GFAP+/Sox2+ neural stem cells in the SGZ. Scale bar: 25 μm. **(G)** Lack of NgR1 expression by DCX+ immature neurons. Scale bars: 25 μm; 10 μm (inset). **(H)** Lack of NgR1 expression by GFAP+ neural stem cells in the SGZ. Scale bar: 25 μm. ML, molecular layer; GCL, granule cell layer; PL, polymorphic layer.

To determine whether neural precursor cells in the subgranular zone and granule cell layer (GCL) of the DG may be potential direct cellular targets of anti-Nogo-A immunotherapy, we performed double-label immunofluorescent staining using the Nogo-A-specific antibody 11C7 and antibodies to cell type-specific markers (Figures 1, 2). Strong Nogo-A expression was found in immature (doublecortin [DCX]-positive) neurons in various stages of development. Both radially-oriented (more mature) cells with more complex arborizations (Figure 2A) and tangentially-oriented (transitioning, less mature progenitors) (Figure 2B; Kempermann et al., 2004) were positive for Nogo-A. Nogo-A expression was especially enriched in the apical dendrites of radially-oriented DCX+ cells.

In contrast, Nogo-A expression by mature dentate granule cells within the GCL was not appreciable by immunofluorescence (**Figrue 2C**), consistent with a previous report (Huber et al., 2002), suggesting transient expression of Nogo-A during the development of adult-born dentate granule cells. Strong Nogo-A expression was observed in large, pyramidal NeuN+ cells at the GCL/polymorphic layer interface (putative basket cells) (Figure 2C, arrowhead), while Nogo-A was not detectable in GFAP+ putative stem cells or astrocytes of the subgranular zone (Figure 2D).

A recently identified receptor for the Nogo-A Δ20 domain, sphingosine-1-phosphate receptor 2 (Kempf et al., 2014), was found to be widely expressed in the DG GCL (as reported by Akahoshi et al., 2011), including in the cell bodies of DCX+ cells (Figure 2E, top and bottom panels). S1PR2 staining typically did not occupy the entirety of the DCX+ cell bodies, possibly suggesting targeting to distinct subcellular domains. Mature NeuN+ granule cell bodies were likewise positive for S1PR2 (Figure 2E, bottom panel; arrowheads: additional DCX/S1PR2 co-expression). GFAP+/Sox2+ cells located in the subgranular zone (putative stem cells) appeared to be weakly S1PR2 positive relative to the stronger S1PR2 expression seen in mature dentate granule cells (Figure 2F).

Lastly, we examined the expression of the Nogo-66 receptor NgR1 in hippocampal neural precursor cells. Throughout the dentate GCL and SGZ, NgR1 expression was observed primarily in a punctate pattern, with more distinctly labeled cell bodies less frequently seen. We found no clear evidence of NgR1 expression by either DCX+ immature neurons or GFAP+ astrocytes or stem cells in the SGZ (Figures 2G,H).

### Lesion size is not affected by antibody treatment

**Figure 3:**
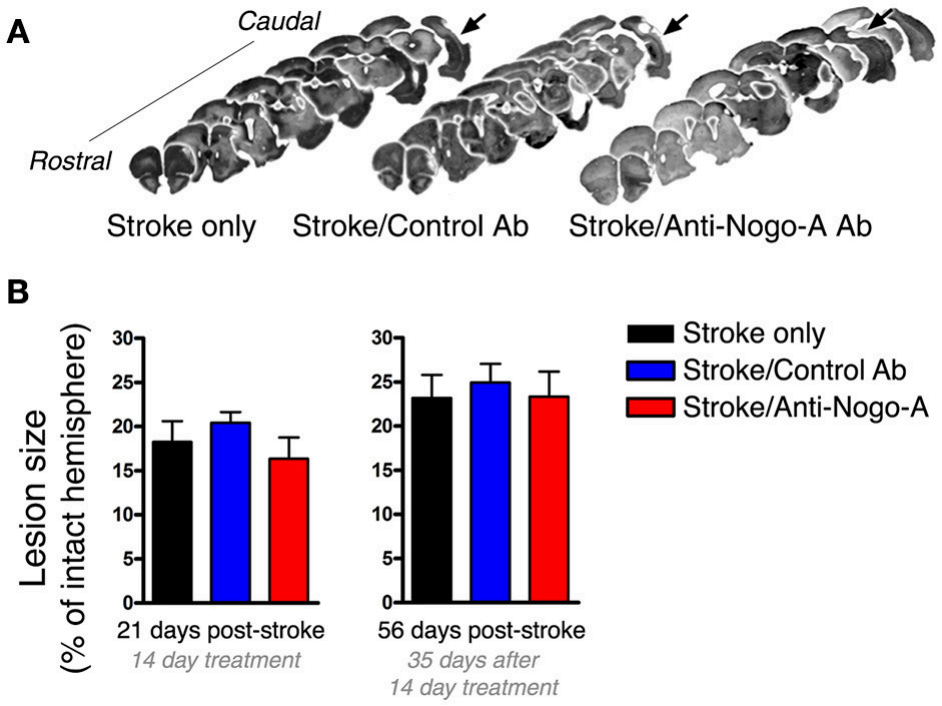
**Lesion size is not affected by antibody treatment. (A)** Representative images of lesion (arrows) location and size from each experimental group at 8 weeks post-stroke. **(B)** Lesion size did not differ among groups at either 21 or 56 days post-stroke.

As the size of the stroke lesion may affect neurogenesis, we measured lesion sizes at both 21 and 56 days post-stroke. Stroke lesions in all experimental groups were unilateral and similar in location, encompassing the dorsolateral cortex and extending from primary motor cortex rostrally through auditory and visual cortices caudally (Figure 3A). Little to no infarction of the underlying white matter or subcortical structures was evident, consistent with previous observations using this model (Gillani et al., 2010). At all-time points, the hippocampus was grossly intact upon brain cryosectioning, but occasionally appeared distorted on the side ipsilateral to the stroke lesion, possibly due to distention of the cerebral ventricles. Lesion sizes were not different among the three treatment groups at either time point assessed (Figure 3B).

### Infused treatment antibody penetrates the hippocampus

**Figure 4:**
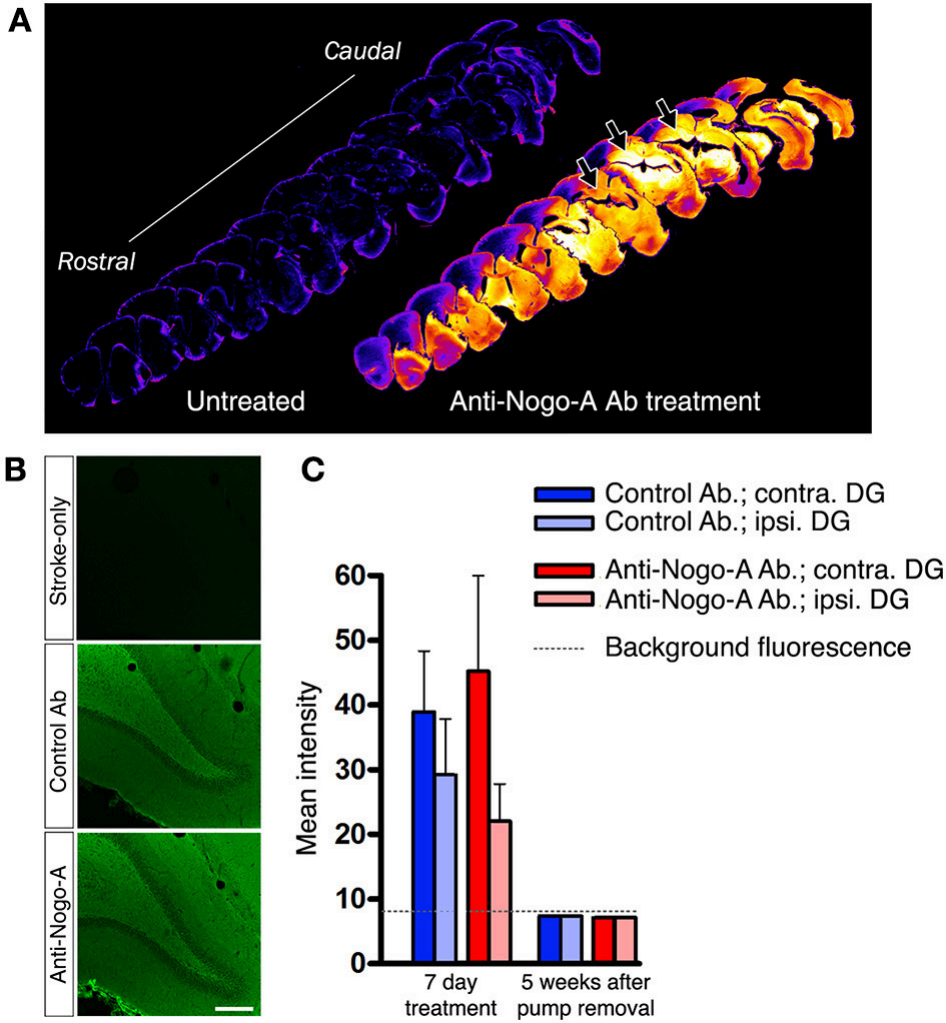
**Infused treatment antibody penetrates hippocampus. (A)** Low magnification images of anti-mouse IgG immunostaining in a stroke-only, untreated subject (left; negative control) and an anti-Nogo-A-treated subject after 3 days of treatment (right). Staining intensities have been remapped, where brighter/hotter colors represent increased signal intensity. Abundant treatment antibody can be seen in the hippocampus (arrows). **(B)** Immunofluorescence staining for mouse IgG after 3 days of antibody infusion shows diffuse, uniform penetration of anti-Nogo-A and control antibodies in the dentate gyrus (DG), whereas only background fluorescence is evident in untreated MCAO rats. Scale bar: 200 μm. **(C)** Quantification of mean fluorescence intensity shows expected elevated signal intensity after 7 days of treatment, whereas at 5 weeks after treatment cessation, the signal is not detectable above background fluorescence by direct immunofluorescence. Contra., contralesional; Ipsi., ipsilesional; DG, Dentate gyrus.

Treatment antibodies penetrated into the hippocampal parenchyma as assessed by immunostaining for mouse IgG after 3 days of treatment (Papadopoulos et al., 2002; Weinmann et al., 2006; Tsai et al., 2007; Figures 4A,B). Treatment antibody was detected in the hippocampus after 3, 7, and 14 days of treatment. Five weeks after pump removal (7 weeks after treatment initiation), both control and anti-Nogo-A antibodies appeared to have been substantially cleared, and were no longer detectable by immunofluorescence above background levels in the DG (Figure 4C).

### Anti-Nogo-A treatment does not alter cellular proliferation in the subgranular zone (SGZ)

**Figure 5:**
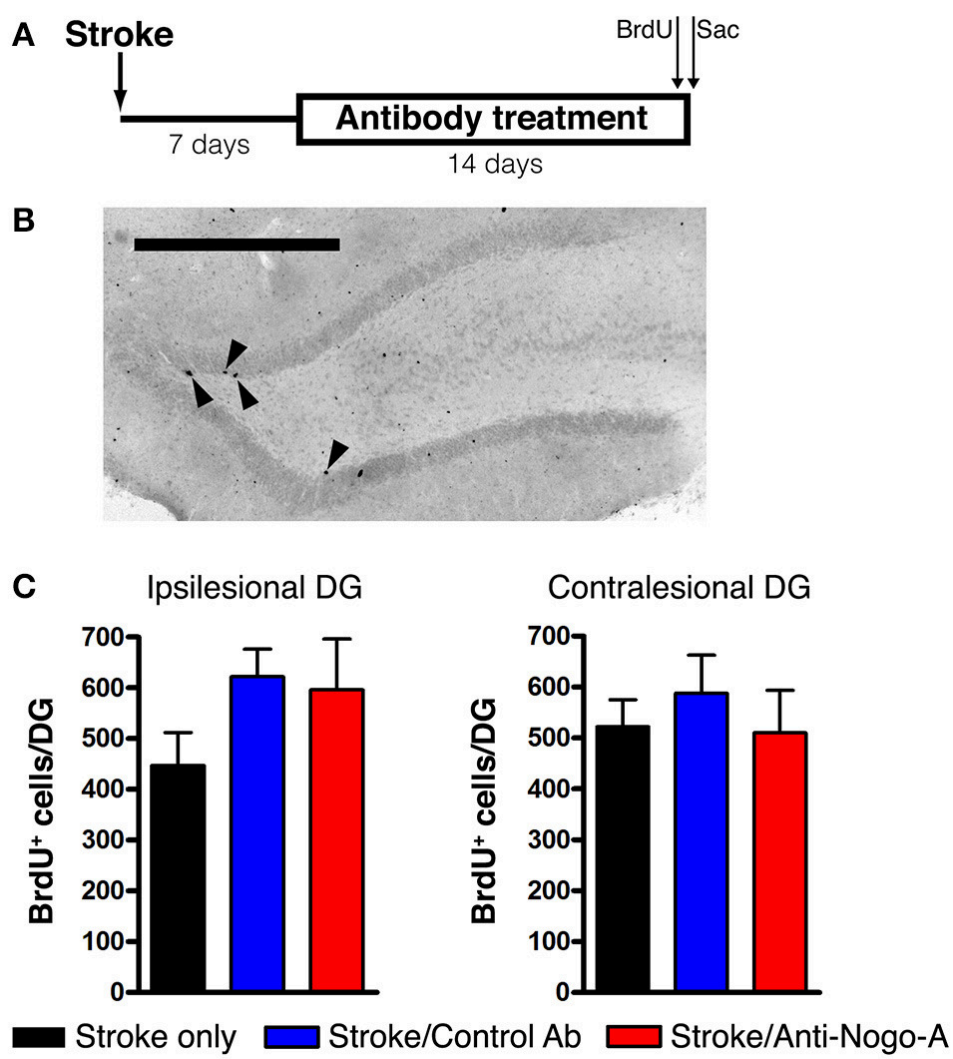
**Cellular proliferation in the subgranular zone and basal granule cell layer is not altered by anti-Nogo-A treatment after stroke. (A)** Overview of BrdU injection strategy to measure cellular proliferation. **(B)** Representative image of BrdU+ nuclei (arrowheads) in the ipsilesional DG of a stroke-only subject at 21 days post-stroke. Scale bar: 500 μm. **(C)** Total numbers of BrdU+ nuclei in the SGZ and basal GCL of the ipsilesional (left) and contralesional (right) DG. Error bars indicate SEM.

Cellular proliferation was measured after 14 days of treatment (i.e., at 21 days post-stroke) by injecting rats with a single dose of BrdU and euthanizing 2 h later (Figures 5A,B). The number of proliferating cells in anti-Nogo-A-treated subjects was not significantly different vs. stroke-only or control antibody-treated controls in either the ipsilesional or contralesional SGZ (Figure 5C; Table 3).

**Table 3:** **Cellular proliferation in the subgranular zone after stroke and anti-Nogo-A immunotherapy**.

**Group**	**Ipsilesional DG**	**Contralesional DG**
Stroke only (*n* = 6)	447 ± 65	522 ± 53
Stroke/Control Ab (*n* = 6)	622 ± 55	588 ± 75
Stroke/Anti-Nogo-A Ab (*n* = 6)	596 ± 100	510 ± 84

Data are presented as mean ± SEM (cells/SGZ).

### Both control antibody and Anti-Nogo-A antibody treatment stimulate the accumulation of new microglia/macrophages, but not new neurons, in the dentate granule cell layer (GCL)

**Figure 6:**
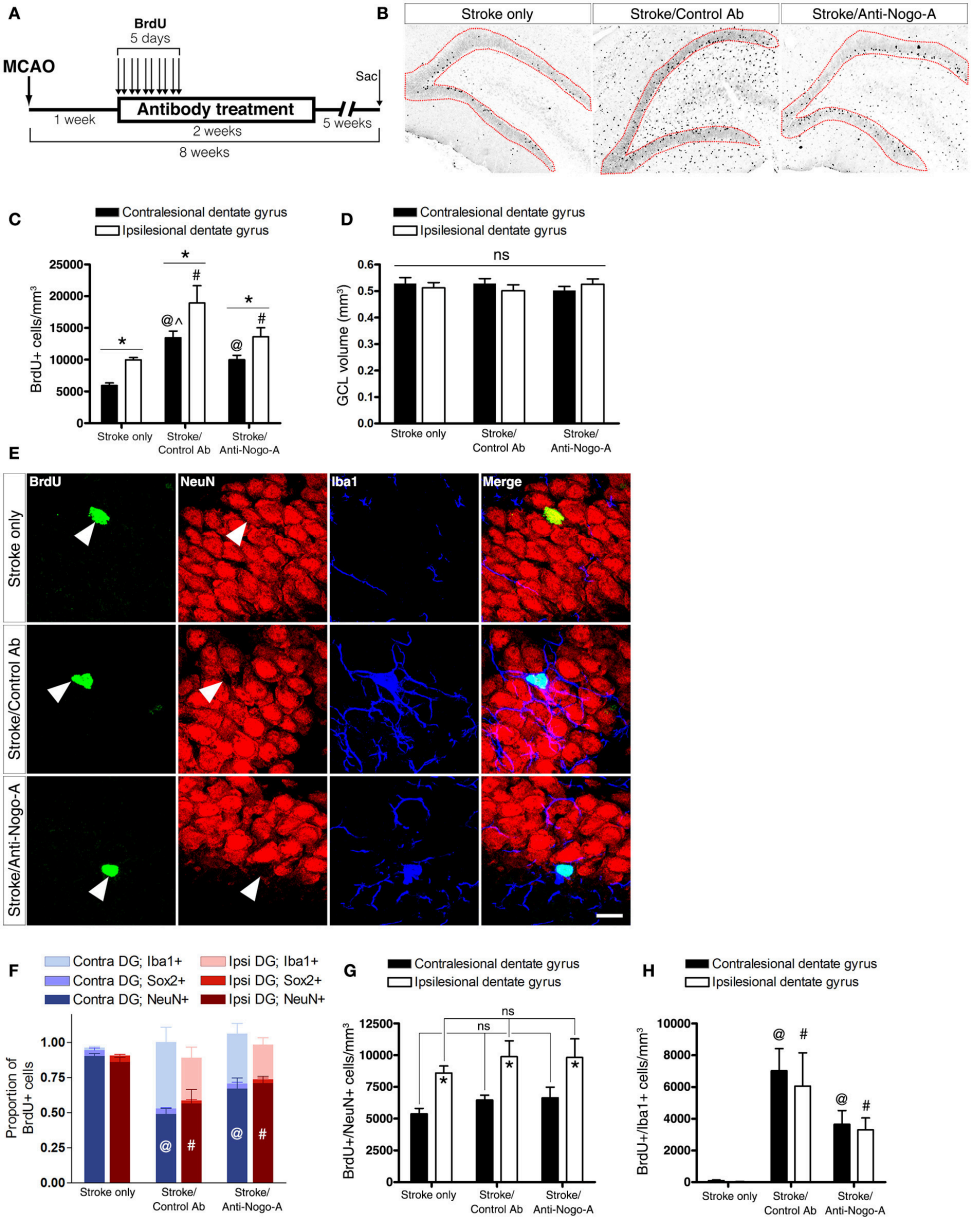
**Both anti-Nogo-A and control antibody treatment induce long-lasting accumulation of new microglia/macrophages without altering neurogenesis. (A)** Overview of BrdU injection strategy to measure the differentiation and survival of proliferating cells. **(B)** Representative images of BrdU immunoreactivity in the ipsilesional dentate gyrus at 8 weeks post-stroke. Newborn cells are evident throughout the DG in both control and anti-Nogo-A antibody groups. The granule cell layer, where cells were counted, is outlined in red. **(C)** Total BrdU+ nuclei in the contralesional (black bars) and ipsilesional (white bars) GCLs. ^*^p < 0.05, ipsilesional vs. contralesional DG (within treatment group); ^@^*p* < 0.05, vs. stroke-only contralesional DG; ^#^*p* < 0.05, vs. stroke-only ipsilesional DG; ^∧^*p* < 0.05, vs. stroke/anti-Nogo-A contralesional GCL. **(D)** The volume of the GCL in which BrdU+ nuclei were counted (in panel “**C**”) was not significantly different among groups. **(E)** Representative image of newborn neurons (BrdU+/NeuN+) and microglia/macrophages (BrdU+/Iba1+) in the GCL. Scale bar: 10 μm. **(F)** Proportions of newborn cells of each phenotype (neuron [NeuN+], microglia/macrophage [Iba1+], neural stem/progenitor cell or astrocyte [Sox2+]). ^@^*p* < 0.05, vs. stroke-only contralesional DG NeuN+ proportion; ^#^*p* < 0.05, vs. stroke-only ipsilesional DG NeuN+ proportion. **(G)** Total number of new neurons in the GCL. ^*^p < 0.05, ipsilesional vs. contralesional DG (within treatment group). **(H)** Total number of new Iba1+ microglia/macrophages in the GCL. ^@^*p* < 0.05, vs. stroke-only contralesional DG; ^#^*p* < 0.05, vs. stroke-only ipsilesional DG. All error bars indicate SEM.

To analyze the phenotypes of newborn cells in the GCL, rats were administered multiple injections of BrdU beginning 7 days after stroke and euthanized for analysis 7 weeks thereafter (Figure 6A). Stroke itself led to a significant increase in the total number of BrdU-positive cells (i.e., cells that had proliferated between days 7 and 11 post-stroke and survived approximately 6–7 weeks thereafter) in the ipsilesional vs. the contralesional GCL (Figures 6B,C; Supplementary Table 2). In both control antibody and anti-Nogo-A treatment groups, more BrdU+ cells were found in the GCL compared to the stroke-only group, but were also more generally distributed throughout the DG (Figures 6B,C). As in the stroke-only group, both antibody-treated groups showed higher numbers of BrdU+ cells in the ipsilesional vs. contralesional GCL (Figure 6C). However, the proportion of BrdU+ cells co-labeled for NeuN (i.e., new neurons) in the GCL was lower in antibody-treated groups (Figure 6F; Supplementary Table 3), such that the total numbers of newborn neurons was not statistically different among groups (Figure 6G; Table 4).

**Table 4:** **Total numbers of newborn neurons in the dentate granule cell layer (GCL) at 8 weeks post-stroke**.

**Group**	**Ipsilesional DG**	**Contralesional DG**
Stroke-only (*n* = 8)	8604 ± 544	5375 ± 434
Stroke/Control Ab (*n* = 5)	9884 ± 1250	6468 ± 383
Stroke/Anti-Nogo-A Ab (*n* = 8)	9822 ± 1463	6636 ± 849

Data are presented as mean ± SEM (new neurons/mm^3^ GCL).

The total volume of the GCL in the area of cell counting in each subject was estimated using Cavalieri's principle. No significant differences were found either among groups or within groups between the ipsilesional and contralesional GCLs when compared at 8 weeks post-stroke (Figure 6D).

Nearly all BrdU+/NeuN– cells in both control antibody and anti-Nogo-A treatment groups were positive for Iba1 (Figures 6E,H; Supplementary Table 4), identifying these cells as microglia/macrophages. Resident microglia were found in the DG of untreated rats, as previously reported (reviewed by Gemma and Bachstetter, 2013), but only rarely had incorporated BrdU.

At 8 weeks post-stroke, only a small percentage (approximately 2–5%) of BrdU+ cells in each group was positive for Sox2, which labels neural stem cells, early intermediate progenitors, and mature astrocytes in the adult rat brain (Komitova and Eriksson, 2004; Yu et al., 2014).

## Discussion

Anti-Nogo-A immunotherapy improves spatial memory after stroke in aged rats, but a cellular mechanism of efficacy has not been identified (Gillani et al., 2010). This study was conducted to determine whether Nogo-A neutralization enhances post-stroke neurogenesis in the dentate gyrus.

We first performed multiple-label immunofluorecent staining to determine whether Nogo-A is expressed by neural precursor cells in the adult DG, thereby identifying possible direct treatment targets. Nogo-A was found to be expressed by doublecortin (DCX)-positive immature neurons, but not stem cells or mature dentate granule cells. To our knowledge, this is the first report of Nogo-A expression in immature neurons of the adult dentate gyrus. This transient expression suggests a stage-specific role of Nogo-A expression in adult hippocampal neuronal development, similar to what has been reported in the adult subventricular zone (Rolando et al., 2012) and during embryonic and early post-natal development (Huber et al., 2002; Aloy et al., 2007; Mingorance-Le Meur et al., 2007; Mathis et al., 2010; Schwab, 2010). Notably, per many of these reports, Nogo-A is expressed by migratory neurons. While we do not directly address the normal physiological role of cell surface and/or intracellular Nogo-A in DG neurogenesis here, we may infer from these previous studies that Nogo-A could play a role in migration of neuronal precursors in the adult DG (Deng et al., 2010; Sun et al., 2015) or in the morphogenesis of new DG neurons (Petrinovic et al., 2013; Kurowska et al., 2014).

Expression of the recently identified receptor for the Nogo-A Δ20 domain, S1PR2, was broadly observed in the dentate granule cell layer, including by immature (DCX+) and mature dentate granule cells. Qualitatively, staining appeared to be stronger in the mature granule cells. Therefore, it is possible that upregulation of S1PR2 begins at the immature neuron stage and persists throughout maturation. Furthermore, we found evidence of S1PR2 expression in GFAP+/Sox2+ cells in the SGZ (putative neural stem cells).

In contrast, NgR1 expression was not clearly seen in neural precursors in the SGZ/DG. We observed punctate NgR1 labeling throughout the GCL, possibly indicative of synaptic localization, as NgR1 has been localized both pre- and post-synaptically (Lee et al., 2008). Cell bodies labeled distinctly for NgR1 were seen infrequently in the GCL, but these cells did not co-express either DCX or GFAP, suggesting against NgR1 expression by immature neurons or neural stem cells.

Examining treatment antibody distribution, we showed that intracerebroventricularly infused antibody entered the hippocampal parenchyma, but was undetectable by immunofluorescence 5 weeks after cessation of treatment. Therefore, direct exposure of target tissue to infused antibody is transient. As we did not analyze antibody distribution at earlier time points after treatment cessation, we cannot conclude that complete antibody clearance requires the full 5 weeks. However, our findings are in line with a previous report noting a reduction in anti-Nogo-A antibody in the brain parenchyma just 1 week after the end of treatment (Marklund et al., 2007). These results raise the possibility that rapid clearance of the antibody from the brain may limit the full potential of anti-Nogo-A antibodies to promote functional recovery, and that a longer treatment duration may be further clinically beneficial.

Despite Nogo-A expression by DCX+ immature neurons (as seen in fixed tissue sections; Figure 2A), we were unable to discern by immunofluorescent histology whether the infused anti-Nogo-A treatment antibody had bound to this cell type *in vivo*. Therefore, it is unclear whether Nogo-A is expressed at the surface of immature neurons in the DG. While Nogo-A is expressed both intracellularly and at the surface of several cell types, including oligodendrocytes and dorsal root ganglion neurons (Caroni and Schwab, 1988; Dodd et al., 2005), Nogo-A was found to be intracellular in a human dopaminergic neuron line (Kurowska et al., 2014). Therefore, subcellular localization of Nogo-A may be cell type-specific. Regardless, intracellular localization of Nogo-A in DCX+ immature neurons would not preclude an indirect effect of anti-Nogo-A treatment antibody on their function. For example, antibody neutralization of surface Nogo-A could block Nogo-A signaling to immature neurons from neighboring cells or myelin.

After inducing a large cortical stroke followed 1 week later by 2 weeks of antibody treatment, we measured the number of proliferating cells in the SGZ. Anti-Nogo-A treatment did not significantly alter the number of proliferating cells in either the ipsilesional or contralesional subgranular zone (SGZ) and basal granule cell layer (GCL) of the dorsal DG. Furthermore, we found no evidence of earlier, transient effects after either 3 or 7 days of treatment (data not shown), suggesting that neural precursor proliferation is unaffected by surface Nogo-A neutralization.

We then investigated the types of cells that were produced after stroke and survived long-term. We found that the proportion of long-lived newborn cells that were positive for NeuN (i.e., new neurons) was approximately 86–90% in the stroke-only group, similar to findings in a previous report (Kluska et al., 2005). Given the increase in total BrdU+ cells in the ipsilesional GCL, this indicates a significant increase in the number of new neurons in the ipsilesional vs. contralesional DG. This result is consistent with reports of increased hippocampal neurogenesis in numerous animal models of stroke, including transient global ischemia (Liu et al., 1998; Kee et al., 2001), transient middle cerebral artery occlusion (Jin et al., 2001; Zhu et al., 2003, 2004), photothrombotic cortical stroke (Kluska et al., 2005), and distal middle cerebral artery occlusion (Matsumori et al., 2006). In contrast, only a small number of BrdU+ cells at 8 weeks post-stroke were Sox2-positive, indicating relatively scant production of new, long-lived neural stem cells and astrocytes.

Both control antibody- and anti-Nogo-A-treated groups exhibited robust accumulation of new Iba1-positive microglia/macrophages in the GCL. In contrast, newborn microglia/macrophages were found very rarely in the GCL of stroke-only subjects. Several potential mechanisms behind the observed accumulation of new microglia/macrophages may be considered. First, the absence of differences in lesion size between treated and untreated groups argues against a direct effect of the lesion itself. Cannulae for antibody delivery are implanted in the lateral cerebral ventricle, and may in rare cases puncture the hippocampal fimbria. However, the fact that BrdU+ cells were generally elevated bilaterally and more uniformly distributed, rather than clustered around a cannula track, makes it unlikely that the observed response was a reaction to mechanical injury. On the other hand, infusion of mouse antibody into the rat CNS could potentially induce a microglial/macrophage response through either recognition of the antibody as a foreign protein, or binding and activation of microglia/macrophage-expressed Fc receptors. Antibody immunogenicity in human patients should be reduced by the use of human antibodies (Nelson et al., 2010), which are currently in use in anti-Nogo-A clinical trials for spinal cord injury and have so far shown an encouraging safety profile (Zörner and Schwab, 2010). While to our knowledge direct demonstration of rat FcR-mouse IgG binding has not been demonstrated, cross-species FcR binding has been reported between more phylogenetically distant species (Lubeck et al., 1985), and FcR cross linking has been shown to stimulate macrophage proliferation (Luo et al., 2010).

The mechanism responsible for improved spatial memory after stroke and anti-Nogo-A treatment is not yet fully understood. While our previous work did not find an effect of anti-Nogo-A treatment on dendritic complexity in CA1, CA3, or DG GCL neurons, a subsequent study noted dendritic alterations in these subfields after acute treatment of hippocampal slice cultures with anti-Nogo-A antibody (Zagrebelsky et al., 2010). These changes were evident after just 4 days of antibody treatment, a much shorter time course than in our previous study, in which histological analysis was performed 10 weeks after the end of treatment. Therefore, it is possible that *in vivo* anti-Nogo-A antibody treatment after stroke leads to rapid changes in dendritic growth that may be pruned back over time (Andres et al., 2011).

Intriguingly, several studies have shown that Nogo-A and its receptors NgR1 and S1PR2 can regulate cognitive function and synaptic plasticity. Transgenic Nogo-A knockdown rats exhibit subtle spatial memory deficits in certain tasks (Petrasek et al., 2014a,b), while mice overexpressing NgR1 show impaired spatial memory performance in the Morris water maze (Karlsson et al., 2016), suggesting that the proper balance of Nogo-A signaling, including during development, is necessary for optimal cognitive function. These effects may also depend on whether Nogo-A signaling perturbation is chronic (as in the case of Nogo-A- or NgR1-transgenic animals), or acute (after neutralizing antibody or blocking peptide treatment). For example, CA3-CA1 long-term potentiation (LTP) was unaffected by null mutation of NgR1 (in the absence of FGF2) (Lee et al., 2008), whereas acute application of an NgR1 blocking antibody enhanced LTP (Delekate et al., 2011). However, both Nogo-A knockdown rats (Tews et al., 2013) and acute hippocampal slices treated with anti-Nogo-A antibodies (Delekate et al., 2011; Kellner et al., 2016) exhibited enhanced CA3-CA1 LTP, suggesting different roles of the ligand (Nogo-A) and receptor (NgR1) in the proper development and function of hippocampal circuitry. Given these findings, it is possible that Nogo-A neutralization improves spatial memory after stroke through a mechanism involving enhanced synaptic plasticity.

Lastly, other properties related to newborn neuron function that we did not examine, including connectivity, synaptogenesis, or morphogenesis, rather than the total number of newborn neurons, may be altered by Nogo-A neutralization. The Nogo receptor NgR1 negatively regulates synaptogenesis and dendritic complexity during hippocampal development (Wills et al., 2012), raising the possibility of a similar role in adult hippocampal neurogenesis. Future studies examining these changes in adult-born neurons after anti-Nogo-A treatment may be enlightening.

In conclusion, our results suggest that anti-Nogo-A immunotherapy does not significantly alter hippocampal neurogenesis after focal cortical stroke in adult rats. We cannot rule out that treatment may induce differences in neurogenesis specifically in aged rats, which were used in our previous study showing efficacy of anti-Nogo-A treatment in improving spatial memory after stroke. However, the present results suggest that different mechanisms outside of enhanced neurogenesis are more likely to underlie this recovery. These results add to our understanding of the scope and limitations of anti-Nogo-A immunotherapy, which are vitally important as anti-Nogo-A antibodies continue to be used in human clinical trials.

## Author contributions

Designed experiments: DS, GK. Performed experiments: DS, ST, RF. Analyzed and interpreted data: DS, ST, TO, GK. Wrote the manuscript: DS, ST, TO, RF, GK.

## Funding

This work was supported by grant 5I01RX000828 from the US Department of Veterans Affairs Rehabilitation Research and Development service to GK, and American Heart Association 15PRE24470136 to DS.

### Conflict of interest statement

The authors declare that the research was conducted in the absence of any commercial or financial relationships that could be construed as a potential conflict of interest.
