# Supplementary material for: Anti-Nogo-A Immunotherapy Does Not Alter Hippocampal Neurogenesis after Stroke in Adult Rats
Source: Front Neurosci. 2016 Oct 18;10:467. doi: 10.3389/fnins.2016.00467 (PMC5067305; doi:10.3389/fnins.2016.00467)
Supplement: Supplementary file 1 [file DataSheet1.docx]

**Supplemental Table 1.** Summary of statistical tests.

|  | **Test** | **Result** | **Significant** |
| --- | --- | --- | --- |
| **Fig 3B (lesion analysis)** | | | |
| 21 days post-stroke; comparison across groups | Unequal variance ANOVA | F(2,16)=1.22  p=0.336 | No |
| 56 days post-stroke; comparison across groups | Unequal variance ANOVA | F(2,18)=0.17  p=0.846 | No |
| **Fig 5C (proliferation)** | | | |
| Contralesional side; comparison across groups | One-way ANOVA | F(2,15)=0.34  p=0.716 | No |
| Ipsilesional side; comparison across groups | One-way ANOVA | F(2,15)=1.55  p=0.244 | No |
| **Fig 6C (total BrdU+ cells at 8 weeks post-stroke)** | | | |
| Stroke only: Contra vs ipsi | Paired t-test | t=-18.61  p<0.001 | Yes |
| Stroke/Control Ab:  Contra vs ipsi | Paired t-test with outlier removed | t=-4.40  p=0.022 | Yes |
| Stroke/Anti-Nogo-A Ab: Contra vs ipsi | Paired t-test | t=-3.63  p=0.008 | Yes |
| Contralesional side; comparison across groups | One-way ANOVA | F(2,18)=27.24  p<0.001 | Yes |
| Contralesional side: Stroke-only vs Stroke/Control Ab | Tukey pairwise comparison | t=-7.24  p<0.001 | Yes |
| Contralesional side: Stroke-only vs Stroke/Anti-Nogo-A Ab | Tukey pairwise comparison | t=-4.47  p=0.001 | Yes |
| Contralesional side: Stroke/Control Ab vs Stroke/Anti-Nogo-A Ab | Tukey pairwise comparison | t=3.32  p=0.010 | Yes |
| Ipsilesional side; comparison across groups | Unequal variance ANOVA | F(2,18)=7.42  p=0.018 | Yes |
| Ipsilesional side: Stroke-only vs Stroke/Control Ab | Kruskal-Wallis with outlier removed | H=8.57  p=0.003 | Yes |
| Ipsilesional side: Stroke-only vs Stroke/Anti-Nogo-A Ab | Pairwise t-test | t=2.47  p=0.043 | Yes |
| Ipsilesional side: Stroke/Control Ab vs Stroke/Anti-Nogo-A Ab | Kruskal-Wallis with outlier removed | H=2.14  p=0.143 | No |
| **Fig 6F (new neuron proportions at 8 weeks post-stroke)** | | | |
| Stroke only: Contra vs ipsi | Paired t-test | t=1.00  p=0.352 | No |
| Stroke/Control Ab:  Contra vs ipsi | Paired t-test | t=1.04  p=0.357 | No |
| Stroke/Anti-Nogo-A Ab: Contra vs ipsi | Paired t-test | t=1.25  p=0.252 | No |
| Contralesional side; comparison across groups | Unequal variance ANOVA | F(2,18)=41.93  p<0.001 | Yes |
| Contralesional side: Stroke-only vs Stroke/Control Ab | Pairwise t-test | t=9.28  p<0.001 | Yes |
| Contralesional side: Stroke-only vs Stroke/Anti-Nogo-A Ab | Pairwise t-test | t=3.01  p=0.020 | Yes |
| Contralesional side: Stroke/Control Ab vs Stroke/Anti-Nogo-A Ab | Pairwise t-test | t=2.15  p=0.057 | No |
| Ipsilesional side; comparison across groups | Unequal variance ANOVA | F(2,18)=5.74  p=0.026 | Yes |
| Ipsilesional side: Stroke-only vs Stroke/Control Ab | Pairwise t-test | t=2.84  p=0.047 | Yes |
| Ipsilesional side: Stroke-only vs Stroke/Anti-Nogo-A Ab | Pairwise t-test | t=2.52  p=0.028 | Yes |
| Ipsilesional side: Stroke/Control Ab vs Stroke/Anti-Nogo-A Ab | Pairwise t-test | t=1.29  p=0.244 | No |
| **Fig 6G (total new neurons at 8 weeks post-stroke)** | | | |
| Stroke only: Contra vs ipsi | Paired t-test | t=6.70  p<0.001 | Yes |
| Stroke/Control Ab:  Contra vs ipsi | Paired t-test | t=2.95  p=0.042 | Yes |
| Stroke/Anti-Nogo-A Ab: Contra vs ipsi | Paired t-test | t=4.15  p=0.004 | Yes |
| Contralesional side; comparison across groups | Unequal variance ANOVA | F(2,18)=1.93  p=0.188 | No |
| Ipsilesional side; comparison across groups | Unequal variance ANOVA | F(2,18)=0.61  p=0.565 | No |
| **Fig 6H (total new microglia/macrophages at 8 weeks post-stroke)** | | | |
| Stroke only: Contra vs ipsi | Wilcoxon Signed Rank | W=6.0  p=0.181 | No |
| Stroke/Control Ab:  Contra vs ipsi | Wilcoxon Signed Rank | W=9.0  p=0.787 | No |
| Stroke/Anti-Nogo-A Ab: Contra vs ipsi | Wilcoxon Signed Rank | W=22.0  p=0.624 | No |
| Contralesional side; comparison across groups | Kruskal Wallis | H(2)=15.22  p<0.001 | Yes |
| Contralesional side: Stroke-only vs Stroke/Control Ab | Pairwise Kruskal Wallis test | H=9.07  p=0.003 | Yes |
| Contralesional side: Stroke-only vs Stroke/Anti-Nogo-A Ab | Pairwise Kruskal Wallis test | H=11.64  p=0.001 | Yes |
| Contralesional side: Stroke/Control Ab vs Stroke/Anti-Nogo-A Ab | Pairwise Kruskal Wallis test | H=2.14  p=0.143 | No |
| Ipsilesional side; comparison across groups | Kruskal Wallis | H=15.43  p<0.001 | Yes |
| Ipsilesional side: Stroke-only vs Stroke/Control Ab | Pairwise Kruskal Wallis test | H=10.13  p=0.001 | Yes |
| Ipsilesional side: Stroke-only vs Stroke/Anti-Nogo-A Ab | Pairwise Kruskal Wallis test | H=12.31  p<0.001 | Yes |
| Ipsilesional side: Stroke/Control Ab vs Stroke/Anti-Nogo-A Ab | Pairwise Kruskal Wallis test | H=1.74  p=0.188 | No |
