# Supplementary material for: Anti-Nogo-A Immunotherapy Does Not Alter Hippocampal Neurogenesis after Stroke in Adult Rats
Source: Front Neurosci. 2016 Oct 18;10:467. doi: 10.3389/fnins.2016.00467 (PMC5067305; doi:10.3389/fnins.2016.00467)
Supplement: Supplementary file 2 [file DataSheet2.docx]

**Supplemental Table 2.** Total BrdU+ cells at 8 weeks post-stroke (mean ± SEM).

| **Group** | **Ipsilesional DG** | **Contralesional DG** |
| --- | --- | --- |
| Stroke only (n=8) | 9958 ± 379 | 5927 ± 424 |
| Stroke/Control Ab (n=5) | 18934 ± 2725 | 13441 ± 1068 |
| Stroke/Anti-Nogo-A Ab (n=8) | 13610 ± 1429 | 9998 ± 692 |

**Supplemental Table 3.** Proportion of NeuN+ new cells at 8 weeks post-stroke (mean ± SEM).

| **Group** | **Ipsilesional DG** | **Contralesional DG** |
| --- | --- | --- |
| Stroke only (n=8) | 0.860 ± 0.032 | 0.903 ± 0.019 |
| Stroke/Control Ab (n=5) | 0.566 ± 0.078 | 0.490 ± 0.032 |
| Stroke/Anti-Nogo-A Ab (n=8) | 0.709 ± 0.051 | 0.671 ± 0.074 |

**Supplemental Table 4.** Total new microglia/macrophages 8 weeks post-stroke (mean ± SEM).

| **Group** | **Ipsilesional DG** | **Contralesional DG** |
| --- | --- | --- |
| Stroke only (n=8) | 22 ± 22 | 92 ± 48 |
| Stroke/Control Ab (n=5) | 6056 ± 2093 | 6578 ± 1599 |
| Stroke/Anti-Nogo-A Ab (n=8) | 3296 ± 752 | 3660 ± 870 |
